# Hexakis(dimethyl sulfoxide-κ*O*)cobalt(III) trinitrate

**DOI:** 10.1107/S1600536809051423

**Published:** 2009-12-09

**Authors:** Qiuhong Li, Seik Weng Ng

**Affiliations:** a College of Materials Science and Engineering, Shandong University of Technology, Zibo 255049, People’s Republic of China; b Department of Chemistry, University of Malaya, 50603 Kuala Lumpur, Malaysia

## Abstract

The metal atom of the title salt, [Co(C_2_H_6_OS)_6_](NO_3_)_3_, is coordinated by six dimethyl sulfoxide mol­ecules in an octa­hedral geometry. The metal atom lies on a special position of 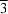 site symmetry. One of the nitrate ions lies on a special position of 3 site symmetry and the other independent ion is disordered about a special position of 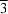 site symmetry.

## Related literature

For the isostructural chromium(III) and iron(III) analogs, see: Öhrström & Svensson (2000 ▶); Tzou *et al.* (1995 ▶).
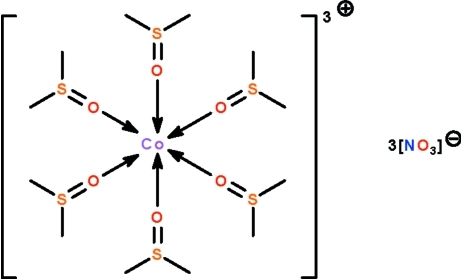


## Experimental

### 

#### Crystal data

[Co(C_2_H_6_OS)_6_](NO_3_)_3_
*M* *_r_* = 713.73
Trigonal, 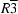

*a* = 11.526 (3) Å
*c* = 19.998 (5) Å
*V* = 2300.8 (10) Å^3^
*Z* = 3
Mo *K*α radiation
μ = 1.03 mm^−1^
*T* = 298 K
0.49 × 0.41 × 0.38 mm

#### Data collection

Bruker SMART 1000 area-detector diffractometer
Absorption correction: multi-scan (*SADABS*; Sheldrick, 1996 ▶) *T*_min_ = 0.632, *T*_max_ = 0.695
3840 measured reflections
1158 independent reflections
879 reflections with *I* > 2σ(*I*)
*R*_int_ = 0.062

#### Refinement

*R*[*F* ^2^ > 2σ(*F* ^2^)] = 0.046
*wR*(*F* ^2^) = 0.136
*S* = 1.07
1158 reflections
67 parameters
7 restraints
H-atom parameters constrained
Δρ_max_ = 0.59 e Å^−3^
Δρ_min_ = −0.62 e Å^−3^

### 

Data collection: *SMART* (Bruker, 1996 ▶); cell refinement: *SAINT* (Bruker, 1996 ▶); data reduction: *SAINT*; program(s) used to solve structure: *SHELXS97* (Sheldrick, 2008 ▶); program(s) used to refine structure: *SHELXL97* (Sheldrick, 2008 ▶); molecular graphics: *X-SEED* (Barbour, 2001 ▶); software used to prepare material for publication: *publCIF* (Westrip, 2009 ▶).

## Supplementary Material

Crystal structure: contains datablocks I, global. DOI: 10.1107/S1600536809051423/ci2949sup1.cif

Structure factors: contains datablocks I. DOI: 10.1107/S1600536809051423/ci2949Isup2.hkl

Additional supplementary materials:  crystallographic information; 3D view; checkCIF report

## Figures and Tables

**Fig. 1.**
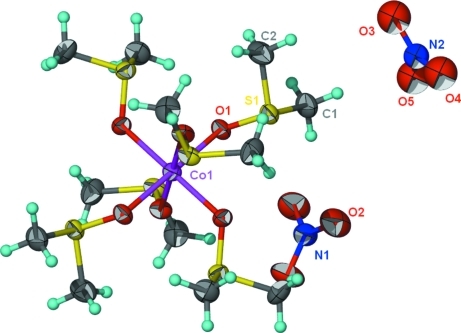
Displacement ellispoid plot (Barbour, 2001) of hexakis(dimethylsulfoxide)cobalt(III) trinitrate at the 50% probability level.

## supplementary crystallographic information

### Experimental

To a solution of cobalt(II) nitrate hexahydrate (0.10 g, 0.4 mmol) in methanol (10 ml) was added a solution of 1,2-disalicyloylhydrazine (0.05 g, 0.2 mmol) in DMSO (10 ml). The red solution was allowed to stand for one week, whereupon red block-shaped crystals were obtained in 60% yield (m.p. > 573 K). CH&N elemental analysis for C_12_H_36_CoN_3_O_15_S_6_: Calculated: C 20.19, H 5.08, N 5.89%; found: C 20.10, H 5.17, N 5.81%.

### Refinement

The nitrate anion lying on the Wyckoff 3*b* position is disordered. This was refined off the special position as a planar four-atom species, with N–O distances restrained to 1.24 (1) Å and O···O distances restrained to 2.15 (1) Å. The isotropic displacement parameters of the three O atoms were restrained to be identical; the O and N atoms were refined isotropically. The methyl H-atoms were placed in idealized positions and treated as riding on their parent atoms with a C–H distance of 0.96 Å [*U*_iso_(H) = 1.5*U*_eq_(C)].

### Crystal data

**Table:**

[Co(C_2_H_6_OS)_6_](NO_3_)_3_	*D*_x_ = 1.545 Mg m^−^^3^
*M**_r_* = 713.73	Mo *K*α radiation, λ = 0.71073 Å
Trigonal, *R*3	Cell parameters from 1446 reflections
Hall symbol: -R 3	θ = 2.3–27.2°
*a* = 11.526 (3) Å	µ = 1.03 mm^−^^1^
*c* = 19.998 (5) Å	*T* = 298 K
*V* = 2300.8 (10) Å^3^	Block, red
*Z* = 3	0.49 × 0.41 × 0.38 mm
*F*(000) = 1116	

### Data collection

**Table:**

Bruker SMART 1000 area-detector diffractometer	1158 independent reflections
Radiation source: fine-focus sealed tube	879 reflections with *I* > 2σ(*I*)
graphite	*R*_int_ = 0.062
φ and ω scans	θ_max_ = 27.4°, θ_min_ = 2.3°
Absorption correction: multi-scan (*SADABS*; Sheldrick, 1996)	*h* = −14→5
*T*_min_ = 0.632, *T*_max_ = 0.695	*k* = −11→14
3840 measured reflections	*l* = −25→25

### Refinement

**Table:**

Refinement on *F*^2^	Primary atom site location: structure-invariant direct methods
Least-squares matrix: full	Secondary atom site location: difference Fourier map
*R*[*F*^2^ > 2σ(*F*^2^)] = 0.046	Hydrogen site location: inferred from neighbouring sites
*wR*(*F*^2^) = 0.136	H-atom parameters constrained
*S* = 1.07	*w* = 1/[σ^2^(*F*_o_^2^) + (0.0617*P*)^2^ + 5.5038*P*] where *P* = (*F*_o_^2^ + 2*F*_c_^2^)/3
1158 reflections	(Δ/σ)_max_ = 0.001
67 parameters	Δρ_max_ = 0.59 e Å^−^^3^
7 restraints	Δρ_min_ = −0.62 e Å^−^^3^

### Special details

**Table:**

Geometry. All e.s.d.'s (except the e.s.d. in the dihedral angle between two l.s. planes) are estimated using the full covariance matrix. The cell e.s.d.'s are taken into account individually in the estimation of e.s.d.'s in distances, angles and torsion angles; correlations between e.s.d.'s in cell parameters are only used when they are defined by crystal symmetry. An approximate (isotropic) treatment of cell e.s.d.'s is used for estimating e.s.d.'s involving l.s. planes.

### Fractional atomic coordinates and isotropic or equivalent isotropic displacement parameters (Å^2^)

**Table:**

	*x*	*y*	*z*	*U*_iso_*/*U*_eq_	Occ. (<1)
Co1	0.6667	0.3333	0.3333	0.0336 (3)	
S1	0.48481 (8)	0.41879 (8)	0.25413 (4)	0.0388 (3)	
C1	0.3940 (4)	0.3538 (5)	0.17922 (18)	0.0567 (10)	
H1A	0.4494	0.3427	0.1470	0.085*	
H1B	0.3673	0.4147	0.1620	0.085*	
H1C	0.3158	0.2686	0.1880	0.085*	
C2	0.3544 (4)	0.4062 (5)	0.3061 (2)	0.0591 (10)	
H2A	0.2805	0.3160	0.3051	0.089*	
H2B	0.3254	0.4662	0.2903	0.089*	
H2C	0.3867	0.4296	0.3511	0.089*	
O1	0.5100 (2)	0.3062 (2)	0.27796 (11)	0.0379 (5)	
O2	0.6649 (4)	0.4393 (3)	0.0814 (2)	0.0838 (11)	
N1	0.6667	0.3333	0.0819 (3)	0.0516 (13)	
N2	0.331 (2)	0.657 (2)	0.1524 (6)	0.060 (4)*	0.1667
O3	0.246 (2)	0.612 (3)	0.1984 (10)	0.104 (5)*	0.1667
O4	0.407 (3)	0.7797 (19)	0.1464 (11)	0.104 (5)*	0.1667
O5	0.338 (2)	0.578 (2)	0.1132 (11)	0.104 (5)*	0.1667

### Atomic displacement parameters (Å^2^)

**Table:**

	*U*^11^	*U*^22^	*U*^33^	*U*^12^	*U*^13^	*U*^23^
Co1	0.0312 (4)	0.0312 (4)	0.0385 (5)	0.01558 (19)	0.000	0.000
S1	0.0334 (5)	0.0343 (5)	0.0474 (5)	0.0159 (4)	−0.0051 (3)	0.0031 (3)
C1	0.057 (2)	0.077 (3)	0.0426 (19)	0.038 (2)	−0.0106 (17)	0.0001 (18)
C2	0.063 (3)	0.069 (3)	0.062 (2)	0.046 (2)	0.000 (2)	−0.009 (2)
O1	0.0327 (12)	0.0352 (12)	0.0462 (12)	0.0173 (10)	−0.0078 (9)	−0.0010 (10)
O2	0.078 (2)	0.0523 (19)	0.130 (3)	0.0389 (18)	0.007 (2)	0.0059 (19)
N1	0.047 (2)	0.047 (2)	0.061 (3)	0.0234 (10)	0.000	0.000

### Geometric parameters (Å, °)

**Table:**

Co1—O1^i^	2.005 (2)	C1—H1C	0.96
Co1—O1^ii^	2.005 (2)	C2—H2A	0.96
Co1—O1^iii^	2.005 (2)	C2—H2B	0.96
Co1—O1^iv^	2.005 (2)	C2—H2C	0.96
Co1—O1	2.005 (2)	O2—N1	1.232 (3)
Co1—O1^v^	2.005 (2)	N1—O2^iv^	1.232 (3)
S1—O1	1.540 (2)	N1—O2^iii^	1.232 (3)
S1—C1	1.765 (4)	N2—O4	1.238 (9)
S1—C2	1.773 (4)	N2—O5	1.238 (9)
C1—H1A	0.96	N2—O3	1.253 (9)
C1—H1B	0.96		
			
O1^i^—Co1—O1^ii^	92.45 (9)	S1—C1—H1B	109.5
O1^i^—Co1—O1^iii^	180.0	H1A—C1—H1B	109.5
O1^ii^—Co1—O1^iii^	87.55 (9)	S1—C1—H1C	109.5
O1^i^—Co1—O1^iv^	87.56 (9)	H1A—C1—H1C	109.5
O1^ii^—Co1—O1^iv^	180.0	H1B—C1—H1C	109.5
O1^iii^—Co1—O1^iv^	92.44 (9)	S1—C2—H2A	109.5
O1^i^—Co1—O1	87.56 (9)	S1—C2—H2B	109.5
O1^ii^—Co1—O1	87.56 (9)	H2A—C2—H2B	109.5
O1^iii^—Co1—O1	92.44 (9)	S1—C2—H2C	109.5
O1^iv^—Co1—O1	92.44 (9)	H2A—C2—H2C	109.5
O1^i^—Co1—O1^v^	92.45 (9)	H2B—C2—H2C	109.5
O1^ii^—Co1—O1^v^	92.44 (9)	S1—O1—Co1	125.03 (13)
O1^iii^—Co1—O1^v^	87.55 (9)	O2^iv^—N1—O2^iii^	119.996 (15)
O1^iv^—Co1—O1^v^	87.56 (9)	O2^iv^—N1—O2	119.995 (10)
O1—Co1—O1^v^	179.998 (1)	O2^iii^—N1—O2	119.996 (15)
O1—S1—C1	103.10 (17)	O4—N2—O5	120.6 (10)
O1—S1—C2	104.99 (17)	O4—N2—O3	120.3 (10)
C1—S1—C2	99.5 (2)	O5—N2—O3	119.0 (10)
S1—C1—H1A	109.5		
			
C1—S1—O1—Co1	151.6 (2)	O1^ii^—Co1—O1—S1	135.7 (2)
C2—S1—O1—Co1	−104.7 (2)	O1^iii^—Co1—O1—S1	−136.90 (12)
O1^i^—Co1—O1—S1	43.10 (12)	O1^iv^—Co1—O1—S1	−44.3 (2)

Symmetry codes: (i) *y*+1/3, −*x*+*y*+2/3, −*z*+2/3; (ii) *x*−*y*+1/3, *x*−1/3, −*z*+2/3; (iii) −*y*+1, *x*−*y*, *z*; (iv) −*x*+*y*+1, −*x*+1, *z*; (v) −*x*+4/3, −*y*+2/3, −*z*+2/3.
